# The effects of Ramadan fasting on clinical and biochemical markers among hemodialysis patients: A prospective cohort study

**DOI:** 10.1371/journal.pone.0218745

**Published:** 2019-06-24

**Authors:** Emad Khazneh, Jamal Qaddumi, Zakaria Hamdan, Falasteen Qudaimat, Asmaa Sbitany, Kamel Jebrin, Osama Sawalmeh, Yousef Abuiram, Mujahed Shraim

**Affiliations:** 1 Nephrology Department, An-Najah National University Hospital, An-Najah National University, Nablus, Palestine; 2 Public Health Department, Faculty of Medicine and Health Sciences, An-Najah National University, Nablus, Palestine; 3 Medicine Department, Faculty of Medicine and Health Sciences, An-Najah National University, Nablus, Palestine; 4 Internal Medicine Department, An-Najah National University Hospital, An-Najah National University, Nablus, Palestine; 5 Public Health Department, College of Health Sciences, Qatar University, Doha, Qatar; University of Mississippi Medical Center, UNITED STATES

## Abstract

**Background:**

Ramadan fasting is compulsory for all healthy adult Muslims. Although sick people are exempted from Ramadan fasting, some patients such as hemodialysis patients prefer to fast during Ramadan. The effect of Ramadan fasting on clinical outcomes and biochemical markers among hemodialysis patients is not clear. The aim of this study was to examine the effects of daily Ramadan fasting and partial Ramadan fasting on key biochemical and clinical markers among hemodialysis patients as compared to hemodialysis patients who chose not to fast during Ramadan.

**Methods:**

A prospective cohort study of 269 end stage renal disease patients were recruited from the hemodialysis unit in An-Najah National University Hospital, Nablus, Palestine. The participants were divided into three cohorts based on their plans for fasting during Ramadan in May 2018; Ramadan fasting group (RFG), Ramadan partial fasting group (RPFG) and Ramadan not-fasting group (RNFG). Key clinical and biochemical markers were measured before, during and after Ramadan.

**Results:**

After adjustment for diabetic and hypertension status and other sociodemographic variables, RFG had higher mean inter-dialytic weight gain (IDWG) by 0.62 kg than RNFG (95% confidence interval (CI) 0.26, 0.99). RPFG also had slight increase in mean IDWG than RNFG by 0.35 kg (95% CI 0.11, 0.60). Additionally, RFG and RPFG had significant increase in mean serum potassium as compared to RNFG. Diabetes was independently associated with increased IDWG by 0.48 kg (0.25, 0.72). Diabetes and hypertension were associated with some independent changes in biochemical markers, but these were clinically negligible.

**Conclusion:**

Our findings suggest that Ramadan fasting (fully or partially) is tolerable by hemodialysis patients and is not associated with important clinical complications. However, these patients should be made aware of the potential risk of fluid overload and hyperkalemia, if they decide to fast during Ramadan. Thus, they should be closely monitored and instructed to adhere to their dietary and fluid intake allowances. Further prospective cohort studies with comprehensive dietary measures and information on adverse clinical outcomes may provide more evidence about the tolerability and safety of Ramadan fasting by hemodialysis patients.

## Introduction

Ramadan is the ninth month of the Islamic lunar calendar during which all healthy adult Muslims are required to abstain from eating any food and drinks from dawn to sunset, with special exceptions for those severely ill, menstruating females, pre-pubertal children and travelers [1–3]. The effect of Ramadan fasting on physiological and biomedical markers among healthy individuals has been widely studied. Research evidence suggests that Ramadan fasting is tolerable and safe for healthy adults [3–9]. Ramadan fasting was found to be associated with beneficial effect on the lipids profile, fasting blood glucose, and body weight among healthy subjects [10–13]. Other studies reported that Ramadan fasting is not associated with significant adverse effects on function of different body systems [14, 15]. Despite the fact that Islam permits sick people and those with significant health problems not to fast [11], many patients still prefer to fast during Ramadan. Several studies have examined the effect of Ramadan fasting on physiological and biomedical markers in patients with kidney diseases. Ramadan fasting was not associated with significant adverse effects in kidney transplant patients after one year of kidney transplantation [16], or urinary risk factors for calculus formation [17]. However, research findings on the safety of Ramadan fasting by patients with chronic kidney disease (CKD) on maintenance hemodialysis are mixed and controversial [18, 19]. Some studies have reported that Ramadan fasting was associated with significant changes in clinical and biomedical markers, such as fluid overload and hyperkalemia, but with no significant complications requiring hospitalization [3, 20]. Other studies found no clinically important variations in biomedical markers in hemodialysis patients during Ramadan fasting [21, 22]. Due to the lack of clear evidence about the safety of Ramadan fasting by CDK and hemodialysis patients, the International Diabetes Federation and Ramadan International Alliance consider these patients to be at very high risk, and are, therefore, exempted from Ramadan fasting. Despite this, the literature suggests that significant proportions of hemodialysis patients still decide to fast during Ramadan. For example, one large multicenter study in Egypt reported that out of 2055 hemodialysis patients 18.5% and 28.4% chose to fast daily or partially during Ramadan in 2016, respectively [23]. The aim of this prospective cohort study was to examine effects of daily Ramadan fasting and partial Ramadan fasting on key biochemical and clinical markers among hemodialysis patients as compared to hemodialysis patients who chose not to fast during Ramadan. We hypothesize that Ramadan fasting is tolerable by hemodialysis patients and is not associated with significant changes in biochemical markers.

## Materials and methods

### Study design, setting, and population

This prospective cohort study was conducted between April and July 2018 at the Dialysis Center of An-Najah National University Hospital, Nablus, Palestine. This hemodialysis unit is the largest dialysis center in the region with more than 320 patients receiving hemodialysis and peritoneal dialysis therapy. All participants were end-stage renal disease patients aged 18 years or older, who were on regular hemodialysis, three times per week with an average of 4 hours per session. Ramadan fasting days in 2018 occurred between May 16^th^, 2018 and June 14^th^, 2018. The average fasting hours was about 16 hours with an average temperature of 27 degrees Celsius. All participants provided written informed consent for participation in the study. The Institutional Review Board of An-Najah National University approved the study.

In April 2018, we invited patients to participate in the study and asked them about their plans for fasting in Ramadan. Then patients who agreed to participate in the study were grouped into three cohorts. The first cohort included those who planned to fast daily during Ramadan (Ramadan Fasting Group (RFG)). The second cohort included those who decided to fast daily during Ramadan excluding hemodialysis days (Ramadan partially fasting group (RPFG)). The third cohort included patients who decided not to fast during Ramadan and continued their dietary habits as usual (Ramadan non-fasting group (RNFG)). The patients were informed to continue their own medications and dietary habits/restrictions during the period of the study.

### Data collection

Demographic and clinical characteristics were collected from patients and their medical records. These included age, gender, number of years on hemodialysis, diabetic status (yes, no), hypertension status (yes, no), inter-dialytic weight gain (IDWG), and education level (primary education, secondary education, college). The biochemical parameters were: serum levels of potassium, phosphorus, albumin, calcium, sodium, blood urea nitrogen (BUN), creatinine, hemoglobin, platelets and erythrocytes count. The biochemical measurements were collected one month prior to Ramadan fasting. The biochemical measurements were then collected during the second week of Ramadan and two weeks after the end of Ramadan. All biochemical parameters were collected before the start of the hemodialysis session on the three data collection occasions. All blood samples were sent to the laboratory for analysis immediately after collection and were analyzed on the same day.

### Statistical analysis

The biochemical and demographic characteristics of the participants were summarized using the descriptive statistics. Means with standard deviation (SD) were used to summarize continuous variables and frequencies with percentages for categorical variables. We used *t*-test and χ^2^ test to examine for any statistically significant differences between the three cohorts on demographic and clinical characteristics. To account for any within individual variations in outcome measures, repeated measures analyses using linear mixed-models were used to examine for any significant differences between the three cohorts in biochemical parameters before, during and after Ramadan. All outcome variables were normally distributed and no data transformation was needed. Any p-value less than 0.05 is considered to be statistically significant and all analyses were conducted using the SPSS computer software version 23.0 (IBM Corp).

## Results

### Baseline characteristics of patients

Two hundred sixty-nine patients were enrolled and all of them completed the study. Thirty-one patients were in the RFG, one-hundred and two patients were in the RPFG, and the remaining one-hundred and thirty-six patients were in the RNFG. All patients completed the study without any changes to their original Ramadan fasting group choice. The mean age of participants was 57.5 years (SD = 13.6) and 151 patients (56.1%) were males. About 45.7% (n = 123) and 41.3% (n = 111) had diabetes and hypertension, respectively. The mean duration on hemodialysis was 3.2 years (SD = 3.7). The baseline demographic and clinical characteristics of patients by Ramadan fasting group are summarized in Table 1. There were no significant differences between Ramadan fasting groups according to age, gender, duration on hemodialysis, and hypertension status. However, there were statistically significant differences between groups according to diabetic status and education level. RNFG had a higher proportion of diabetic patients (58.1%) than RFG (38.7%) and RPFG (31.6%). RFG and RPFG had slightly higher education level than RNFG.

**Table 1:** Baseline demographic and biomedical characteristics of hemodialysis patients by Ramadan fasting group.

Variable	RFG (n = 31)	RPFG (n = 102)	RNFG (n = 136)	P-value
**Age**	56.9 (11.5)	55.2 (14.1)	59.4 (13.4)	0.064
**Gender**				
Female	10 (32.3)	42 (41.2)	66 (48.5)	0.202
Male	21 (67.7)	60 (58.8)	70 (51.5)	
**Education level**				
Primary school	26 (83.9)	76 (74.5)	123 (90.4)	0.009
Secondary school	3 (9.7)	23 (22.5)	11 (8.1)	
College	2 (6.5)	3 (2.9)	2 (1.5)	
**Duration on dialysis (years)**	2.4 (2.6)	3.7 (3.8)	3.0 (3.8)	0.202
**Diabetic Mellitus status**				
No	19 (61.3)	70 (68.4)	57 (41.9)	<0.001
Yes	12 (38.7)	32 (31.6)	79 (58.1)	
**Hypertension status**				
No	18 (58.1)	63 (61.8)	77 (56.6)	0.725
Yes	13 (41.9)	39 (38.2)	59 (43.4)	

Data is either Mean (Standard deviation) or Frequency (%). RFG, Ramadan fasting group; RPFG, Ramadan partial fasting group; RNFG, Ramadan non-fasting group.

### Effect of Ramadan fasting on biochemical parameters

After adjustment for diabetes and hypertension status and other statistically significant demographic variables, RFG had higher mean IDWG by 0.62 kg than RNFG (95% CI 0.26, 0.99; p 0.001). RPFG had slightly higher mean IDWG by 0.23 kg than RNFG, (95% CI 0.11, 0.60; p 0.005). RPFG had increased serum creatinine by 0.77 mg/dl than RNFG (95% CI 0.30, 124; p 0.001), but no significant difference in serum creatinine was found between RFG and RNFG (Table 2). RFG had higher mean serum potassium than RNFG by 0.48 mEq/L (0.26, 0.70; p <0.001), but there was no significant difference in mean serum potassium between RPFG and RNFG.

**Table 2:** Adjusted* effects of Ramadan fasting on clinical and biochemical parameters.

Biochemical variable	Predictor Variable	Effect estimate (95% CI)	SE of effect estimate	Mean (95% CI)	SE of mean	P-value
Inter-dialytic weight gain (kg)	**Intercept**	4.25 (3.70, 4.81)	0.28	-	-	< .001
	**Group**					
	RFG	0.62 (0.26, 0.99)	0.19	3.80 (3.49, 4.15)	0.17	0.001
	RPFG	0.35 (0.11, 0.60)	0.13	3.55 (3.37, 3.74)	0.09	0.005
	RNFG	Ref	-	3.20 (3.04, 3.36)	0.08	
	**Diabetic status**					
	No	Ref	-	3.25 (3.07, 3.43)	0.09	
	Yes	0.48 (0.25, 0.72)	0.12	3.74 (3.56, 3.92)	0.09	<0.001
	**Hypertension status**					
	No	Ref	-	3.45 (3.28, 3.62)	0.08	
	Yes	0.18 (0.05, 0.41)	0.12	3.63 (3.44, 3.82)	0.09	0.123
Albumin g/dl	**Intercept**	4.03 (3.83, 4.22)	0.10	-	-	<0.001
	**Group**					
	RFG	0.05 (-0.08, 0.18)	0.06	3.82 (3.70, 3.93)	0.06	0.448
	RPFG	0.08 (-0.01, 0.17)	0.04	3.85 (3.79, 3.92)	0.03	0.055
	RNFG	Ref	-	3.77 (3.71, 3.82)	0.03	
	**Diabetic status**					
	No	Ref	-	3.87 (3.81, 3.93)	0.03	
	Yes	0.12 (0.04, 0.20)	0.04	3.75 (3.69, 3.82)	0.03	0.004
	**Hypertension status**					
	No	Ref	-	3.77 (3.71, 3.82)	0.03	
	Yes	0.09 (0.01, 0.17)	0.04	3.86 (3.79, 3.92)	0.03	0.026
Creatinine mg/dl	**Intercept**	10.11 (9.05, 11.18)	0.54	-	-	<0.001
	**Group**					
	RFG	0.31 (-0.38, 1.01)	0.35	8.14 (6.99, 9.29)	0.58	0.378
	RPFG	0.77 (0.30, 1.24)	0.24	8.55 (7.91, 9.20)	0.33	0.001
	RNFG	Ref	-	8.11 (7.55, 8.67)	0.28	
	**Diabetic status**					
	No	Ref	-	8.39 (7.76, 9.01)	0.33	
	Yes	-0.52 (-0.97, -0.07)	0.23	8.14 (7.50, 8.78)	0.32	0.023
	**Hypertension status**					
	No	Ref	-	3.87 (3.81, 3.93)	0.03	
	Yes	0.72 (0.28, 1.16)	0.22	3.75 (3.69, 3.82)	0.03	0.001
Blood urea nitrogen mg/dl	**Intercept**	66.42 (59.50, 73.34)	3.51	-	-	<0.001
	**Group**					
	RFG	2.09 (-2.36, 6.54)	2.25	58.51 (54.51, 62.52)	2.04	0.355
	RPFG	2.36 (-0.65, 5.37)	1.53	58.78 (56.49, 61.07)	1.16	0.124
	RNFG	Ref	-	56.42 (54.51, 58.34)	0.97	
	**Diabetic status**					
	Yes	Ref	-	57.53 (55.46, 59.59)	1.05	0.599
	No	0.76 (-3.61, 2.09)	1.45	58.29 (55.95, 60.62)	1.19	
	**Hypertension status**					
	No	Ref	-	55.63 (53.60, 57.65)	1.03	0.001
	Yes	4.56 (1.77, 7.34)	1.42	60.19 (57.85, 62.52)	1.19	
Potassium mEq/l	**Intercept**	5.52 (5.18, 5.85)	0.17	-	-	<0.001
	**Group**					
	RFG	0.48 (0.26, 0.70)	0.11	5.38 (5.18, 5.58)	0.10	<0.001
	RPFG	0.12 (-0.03, 0.27)	0.08	5.03 (4.92, 5.14)	0.06	0.104
	RNFG	Ref	-	4.90 (4.81, 5.00)	0.05	
	**Diabetic status**					
	No	Ref	-	5.10 (5.00, 5.20)	0.05	
	Yes	0.01 (-0.14, 0.15)	0.07	5.11 (4.99, 5.22)	0.06	0.931
	**Hypertension status**					
	No	Ref	-	4.99 (4.89, 5.09)	0.05	
	Yes	0.24 (0.10, 0.38)	0.07	5.22 (5.11, 5.34)	0.06	0.001
Phosphorus mg/dl	**Intercept**	5.62 (4.96, 6.28)	0.33	-	-	<0.001
	**Group**					
	RFG	0.12 (-0.32, 0.55)	0.22	4.86 (4.47, 5.25)	0.20	0.600
	RPFG	-0.11 (-0.40, 0.19)	0.15	4.64 (4.41, 4.86)	0.11	0.471
	RNFG	Ref	-	4.74(4.56, 4.93)	0.10	
	**Diabetic status**					
	No	Ref	-	4.85 (4.65, 5.05)	0.10	
	Yes	-0.20 (-0.48, 0.08)	0.14	4.65 (4.42, 4.87)	0.12	0.154
	**Hypertension status**					
	No	Ref	-	4.57 (4.38, 4.77)	0.10	
	Yes	0.35 (0.08, 0.62)	0.14	4.92 (4.69, 5.15)	0.12	0.012
Calcium mg/dl	**Intercept**	8.33 (7.92, 8.74)	0.21	-	-	<0.001
	**Group**					
	RFG	0.22 (-0.05, 0.49)	0.14	9.13 (8.88, 9.37)	0.12	0.103
	RPFG	0.01 (-0.17, 0.19)	0.09	8.91 (8.77, 9.05)	0.07	0.914
	RNFG	Ref	-	8.90 (8.79, 9.02)	0.06	
	**Diabetic status**					
	No	Ref	-	9.03 (8.91, 9.15)	0.06	
	Yes	-0.09 (-0.27, 0.07)	0.09	8.93 (8.79, 9.07)	0.07	0.263
	**Hypertension status**					
	No	Ref	-	8.92 (8.79, 9.04)	0.06	
	Yes	0.13 (-0.04, 0.30)	0.09	9.04 (8.90, 9.19)	0.07	0.137
Sodium mEq/l	**Intercept**	133.94 (132.09, 135.79)	0.93	-	-	<0.001
	**Group**					
	RFG	-0.72 (-1.92, 0.50)	0.61	136.27 (135.18, 137.36)	0.55	0.247
	RPFG	-0.48 (-1.30, 0.34)	0.42	136.50 (135.88, 137.13)	0.32	0.252
	RNFG	Ref	-	136.98 (136,46, 137.51)	0.27	
	**Diabetic status**					
	No	Ref	-	137.73 (138.17, 138.29)	0.29	
	Yes	-2.29 (-3.08, -1.51)	0.40	135.44 (134.80, 136.08)	0.32	<0.001
	**Hypertension status**					
	No	Ref	-	136.50 (135.95, 137.05)	0.28	
	Yes	0.18 (-0.34, 1.30)	0.42	136.67 (136.04, 137.31)	0.32	0.252
Hemoglobin g/dl	**Intercept**	10.86 (10.51, 11.21)	0.18	-	-	<0.001
	**Group**					
	RFG	0.45 (-0.02, 0.92)	0.24	11.27 (10.84, 11.69)	0.22	0.061
	RPFG	0.20 (-0.12, 0.52)	0.16	11.02 (10.77, 11.26)	0.12	0.217
	RNFG	Ref	-	10.82 (10.61, 11.02)	0.10	
	**Diabetic status**					
	No	Ref	-	10.85 (10.64, 11.07)	0.11	
	Yes	0.36 (0.06, 0.66)	0.15	11.21 (10.96, 11.46)	0.13	0.019
	**Hypertension status**					
	No	Ref	-	11.13 (10.92, 11.35)	0.11	
	Yes	-0.20 (-0.50, 0.09)	0.15	10.93 (10.68, 11.18)	0.13	0.177
Erythrocyte count 1000/μL	**Intercept**	3.78 (3.65, 3.91)	0.07	-	-	<0.001
	**Group**					
	RFG	<0.01 (-0.17, 0.17)	0.09	3.70 (3.54, 3.86)	0.08	0.987
	RPFG	-0.04 (-0.12, 0.52)	0.06	3.66 (3.57, 3.75)	0.05	0.512
	RNFG	Ref	-	3.70 (3.62, 3.77)	0.04	
	**Diabetic status**					
	No	Ref	-	3.63 (3.55, 3.71)	0.04	
	Yes	0.11 (-0.01, 0.22)	0.06	3.74 (3.65, 3.83)	0.05	0.060
	**Hypertension status**					
	No	Ref	-	3.66 (3.58, 3.74)	0.04	
	Yes	0.04 (-0.07, 0.15)	0.06	3.70 (3.61, 3.80)	0.05	0.477
Platelets 1000/μL	**Intercept**	202.78 (186.49, 219.08)	8.28	-	-	< .001
	**Group**					
	RFG	1.64 (-21.71, 24.99)	11.86	205.40 (184.34, 226.36)	10.70	0.890
	RPFG	-5.65 (-21.38, 10.09)	7.99	198.11 (186.11, 210.11)	6.09	0.480
	RNFG	Ref	-	203.76 (193.71, 213.80)	5.10	
	**Diabetic status**					
	No	Ref	-	194.60 (183.85, 205.35)	5.46	
	Yes	-15.65 (-30.35, -0.95)	7.47	210.25 (198.04, 222.46)	6.20	0.037
	**Hypertension status**					
	No	Ref	-	203.86 (193.29, 214.43)	5.37	
	Yes	-2.87 (-11.55, 17.29)	7.32	200.99 (188.79, 213.18)	6.19	0.392

RFG, Ramadan fasting group; RPFG, Ramadan partial fasting group; RNFG, Ramadan non-fasting group; SE, Standard Error; CI, Confidence Interval; Ref, Reference category.

* Effect estimates are adjusted for statistically significant predictors of clinical and biochemical parameters, including age, gender, duration on hemodialysis, and education level.

### Independent effects of diabetes and hypertension on biochemical variables

Diabetic status was a significant predictor of a number changes in biochemical parameters independent of Ramadan fasting. Diabetic patients had high mean IDWG by 0.48 kg than non- diabetic patients (95% CI 0.25, 0.72; p <0.001). Diabetic patients had slightly higher mean albumin by 0.12 g/dl than non-diabetic patients (95% CI 0.04, 0.20; p 0.004). Mean serum creatinine was lower in diabetic patients than non-diabetic patients by 0.52 md/dl (95% CI -0.97, -0.07; p 0.023). Additionally, mean sodium was slightly higher in diabetic patients than non- diabetic patients by 2.29 mEq/l (95% CI -3.08, -1.51; p <0.001). In addition, mean hemoglobin was slightly higher in patients with diabetes than non-diabetic patients by 0.36 g/dl (95% CI 0.06, 0.66; p 0.019). Also, slight decrease in mean platelets by 15.65 1000/μL among diabetic patients as compared to those without diabetes (95% CI -30.35, -0.095; p 0.037). Similarly, hypertensive patients had slight increase in mean albumin, creatinine, urea, potassium, and phosphorus levels than patients without hypertension (Table 2).

## Discussion

The aim of this cohort study was to assess the effects of daily Ramadan fasting and partial Ramadan fasting on key biochemical and clinical markers among hemodialysis patients in comparison to hemodialysis patients who did not fast during Ramadan. After controlling for within person variation, sociodemographic and significant comorbidities (diabetic and hypertension status), we found no clinically significant differences between Ramadan fasting groups in means of the majority of biochemical variables including serum albumin, creatinine, blood urea nitrogen, phosphorus, calcium, sodium, hemoglobin, erythrocytes count, and platelets count. This accord with the findings of prior studies suggesting that, on average, Ramadan fasting by hemodialysis patients is not associated with significant clinical changes in key biochemical parameters [3, 21, 24]. The current study found that RFG and RPFG had slightly higher mean IDWG by 0.6 kg and 0.4 kg than RNFG, respectively. Prior studies have shown mixed findings with respect to the effect of Ramadan fasting on IDWG in hemodialysis patients. One study reported that Ramadan fasting was associated with increase in mean IDWG by 0.6 kg among Saudi hemodialysis patients [20]. Other studies reported that Ramadan fasting was associated with either decrease or no significant difference in IDWG [3, 22, 24, 25]. The underlying mechanisms for our observation of increasing mean IDWG in RFG and RPFG is not clear, as our study did not collect dietary information. However, the increase in IDWG may be explained by increase in fluid intake by patients after breaking their fast and during the night [1, 20]. For example, one study reported that some hemodialysis patients consumed over three liters of water and other beverages from sunset to dawn [20]. Our finding of increased mean IDWG by 0.5 kg in diabetic patients as compared to patients without diabetes, independent of Ramadan fasting status, may be attributed to oral dryness and higher hemoglobin A1c level [26]. The present study found a significant increase in mean serum potassium in RFG than that of RNFG suggesting that hemodialysis patients fasting daily during Ramadan may be at risk of hyperkalemia. Although, this finding differs from some previous studies [3, 21–24], which showed no significant changes in serum potassium due to Ramadan fasting in hemodialysis patients, it is consistent with the findings of another study from KSA by Al-Khader and colleagues [20]. However, no adverse effects related to hyperkalemia were reported by that study [20]. The reason for increase in serum potassium in RFG in the current study is not clear. However, one potential explanation is increased consumption of Ramadan’s festive foods rich in potassium (e.g. dates, apricot, watermelon, sweets, carbonated soft drinks, squash, tea and coffee), which is traditional in Palestine and other middle eastern countries [3, 10, 20, 24]. Another explanation is insulin suppression due to fasting (insuliopenia), causing shift of potassium from the intracellular to the extracellular space [27, 28].

Although the evidence from this study suggests that Ramadan fasting by hemodialysis patients is not associated with clinically significant complications, it highlights that these patients may be at risk of hyperkalemia and fluid overload. Therefore, hemodialysis patients who intend to fast during Ramadan should be made aware of these potential complications. Additionally, these patients should be monitored closely for signs and symptoms of hyperkalemia and fluid overload. They should also be advised to avoid exceeding their daily fluid allowance and limit their intake of food rich in potassium.

### Strengths and limitations

This study included relatively a large number of patients undergoing hemodialysis at An-Najah National University Hospital. The number of hemodialysis patients in this unit represents about 20% of all hemodialysis patients in the West Bank, Palestine [29]. So, the demographic, clinical, and biochemical characteristics of included hemodialysis patients are likely to be generalizable to hemodialysis population in Palestine. Another important strength of the study is that the effects of Ramadan fasting on biochemical markers was estimated using RFG, RPFG, and RNFG cohorts. This information is useful for hemodialysis patients considering full or partial Ramadan fasting. Another strength is that biochemical markers were measured before, during, and after Ramadan. Additionally, we used repeated measured with mixed models to account for within-person variation in biochemical markers, and the findings are independent of key demographic and clinical characteristics of hemodialysis patients. In addition, our study was conducted in the summer (May-June, 2018), when the weather is hot and relatively dry in Nablus region, which provides some reassurance about the tolerability of Ramadan fasting by hemodialysis patients at this time of the year. However, this may not generalize to other regions with longer duration of Ramadan fasting and different temperature and humidity conditions. Another important limitation of our study is that we did not measure or account for potential effects of dietary changes during Ramadan or dietary habits more generally on biochemical markers. One more limitation is that our study did not collect information on some adverse events (such as emergency visits or hospital admissions). However, prior studies have shown that occurrence of such adverse events is low among hemodialysis patients fasting during Ramadan [3, 20, 21]. More research with appropriate dietary measures and information on adverse events among hemodialysis patients fasting during Ramadan is needed to help clinicians provide appropriate and safe recommendations for hemodialysis patients considering full or partial Ramadan fasting.

## Conclusions

This study adds further evidence that Ramadan fasting, fully or partially, is tolerable and is not associated with significant clinical complications in hemodialysis patients. However, these patients are at risk of fluid overload and hyperkalemia. Therefore, they should be closely monitored and instructed to adhere to their daily fluid allowance and limit their consumption of food rich in potassium. Further prospective cohort studies with dietary and adverse events measures may provide clearer evidence about the tolerability and safety of full or partial Ramadan fasting by hemodialysis patients.

## Supporting information

S1 File All data underlying the findings. (XLSX)
